# All-in-one sequencing: an improved library preparation method for cost-effective and high-throughput next-generation sequencing

**DOI:** 10.1186/s13007-020-00615-3

**Published:** 2020-05-24

**Authors:** Sheng Zhao, Cuicui Zhang, Jianqiang Mu, Hui Zhang, Wen Yao, Xinhua Ding, Junqiang Ding, Yuxiao Chang

**Affiliations:** 1 Shenzhen Branch, Guangdong Laboratory for Lingnan Modern Agriculture, Genome Analysis Laboratory of the Ministry of Agriculture, Agricultural Genomics Institute at Shenzhen, Chinese Academy of Agricultural Sciences, Shenzhen, 518120 China; 2 College of Life Science and Technology, Guangxi University, Nanning, 530004 China; 3 National Key Laboratory of Wheat and Maize Crop Science, College of Life Sciences, Henan Agricultural University, Zhengzhou, 450002 China; 4 State Key Laboratory of Crop Biology, College of Plant Protection, Shandong Agricultural University, Taian, 271018 China; 5 National Key Laboratory of Wheat and Maize Crop Science, College of Agronomy, Henan Agricultural University, Zhengzhou, 450002 China

**Keywords:** All-in-one sequencing (AIO-seq), Library preparation, Population genetic research, Whole genome sequencing, RNA-seq

## Abstract

**Background:**

Next generation sequencing (NGS) has been widely used in biological research, due to its rapid decrease in cost and increasing ability to generate data. However, while the sequence generation step has seen many improvements over time, the library preparation step has not, resulting in low-efficiency library preparation methods, especially for the most time-consuming and labor-intensive steps: size-selection and quantification. Consequently, there can be bottlenecks in projects with large sample cohorts.

**Results:**

We have described the all-in-one sequencing (AIO-seq) method, where instead of performing size-selection and quantification for samples individually, one sample one tube, up to 116 samples are pooled and analyzed in a single tube, ‘All-In-One’. The AIO-seq method pools libraries based on the samples’ expected data yields and the calculated concentrations of the size selected regions (target region), which can easily be obtained with the Agilent 2100 Bioanalyzer and Qubit Fluorometer. AIO-seq was applied to whole genome sequencing and RNA-seq libraries successfully, and it is envisaged that it could be applied to any type of NGS library, such as chromatin immunoprecipitation coupled with massively parallel sequencing, assays for transposase-accessible chromatin with high-throughput sequencing, and high-throughput chromosome conformation capture. We also demonstrated that for genetic population samples with low coverage sequences, like recombinant inbred lines (RIL), AIO-seq could be further simplified, by mixing the libraries immediately after PCR, without calculating the target region concentrations.

**Conclusions:**

The AIO-seq method is thus labor saving and cost effective, and suitable for projects with large sample cohorts, like those used in plant breeding or population genetics research.

## Background

The capabilities of high output sequencing and the rapid decreases in its cost, especially with the Illumina Hiseq X Series and NovaSeq Systems, have made whole genome sequencing (WGS) for a large cohort of samples both possible and affordable [1, 2]. The completion of WGS projects has deepened our understanding of many biological mechanisms and given excellent examples as to how NGS technologies have revolutionized biological research in the era of low-cost sequencing. Current trends suggest that there is a scientific need to sequence more samples for research purposes [3], and this is expected to further increase with time; for example, the project MinE requires sequence data for 15,000 amyotrophic lateral sclerosis patients and 7500 matched controls for a comprehensive study of the disease [4]. In addition to the applications of WGS, numerous sophisticated novel NGS-based methods have been developed for genomic research, such as chromatin immunoprecipitation coupled with massively parallel sequencing (ChIP-seq), assays for transposase-accessible chromatin with high-throughput sequencing (ATAC-seq), restriction site associated DNA sequencing (RAD-seq), methyl-seq, and many more [5]. These novel methods have greatly expanded the technologies available to investigate variable genomic phenomena.

Compared with the fact that DNA sequencing costs have decreased more than 380,000-fold since 2001 (http://www.genome.gov/sequencingcostsdata), little has changed in the library preparation methods, creating bottlenecks in sequencing procedures, especially when a large cohort of samples are involved. Processing of the pre-sequencing samples generally includes DNA fragmentation, end-polishing, the ligation of adaptors, limited-cycles of PCR, library size selection and quantification, and overall, is quite labor-intensive and time-consuming. There have consequently been numerous attempts to improve the pre-sequencing methods, to match the dramatic improvements seen in the actual sequencing technologies [6–8]. For example, the replacement of the Klenow fragment (exo-) with Taq polymerase for A-tailing reactions and buffer optimization, enabled all the steps of end-repair, A-tailing, and adaptor ligation, to be conducted in the same tube without DNA purification [7]. Moreover, another significant improvement in the library preparation was the application of transposases such as Tn5 and MuA, which could mediate the fragmentation of the double-strand DNA and the ligation of synthetic oligonucleotides at both ends, in a 5-min single-tube reaction [9–11]. There have also been other innovations to overcome the limitations in the library preparation processes [8]. Indeed, these optimizations, such as the use of specific enzymes, reagents, reaction conditions, as well as the application of novel equipment, have improved the library preparation efficiency and the resulting library quality [8, 12]. However, the steps of library size selection and quantification remain areas of active and fertile research, as they require most of the hands-on time in whole library preparations, but have previously been ignored as targets of optimization. In the early stages of NGS, traditional manual agarose gel excisions after electrophoresis were used for library size selection, then a solid-phase reversible immobilization bead-based method was widely used, as it was suitable to scale up the automated liquid handler for large sample cohorts, but the target region could not be controlled accurately [13, 14]. There are a wide variety of alternatives for automated fragment size selection, such as E-Gel from Thermo Fisher Scientific Incorporated, Labchip XT, and products from Sage Science Incorporated. However, no tool is considered to be perfect, as while each is considered to have outstanding performance for a single factor such as DNA fragment size range and accuracy, recovery efficiency, cost per sample, and so on, the improvement of one of these characteristics generally comes at the expense of another [14]. After size selection, the selected fragments need to be quantified accurately to calculate the amounts required for multiplexing different samples, according to their concentrations and expected sequence yields, as well as the DNA amount for loading onto a sequencing flowcell. Inaccurate library quantification could give large deviations from the expected sequence data for individual sample; as underloading the libraries could reduce cluster density, and hence data yield, while overloading could generate higher cluster density but with low passing filter ratio and qualified data output, and even failure of the whole lane or flowcell. Like the situation for size selection, there are a few options available for library quantification, including quantitative PCR (qPCR), droplet digital PCR (ddPCR), ddPCR-tail, QuantiSize, and quantitative MiSeq [15–17], but none have high-quality performance for all important characteristics, such as accuracy, cost per sample, labor and time, etc.

Despite many attempts to optimize the methods for size selection and quantification, libraries after PCR still need to be size selected and quantified using a one sample one tube method, to get accurate fragment size and library concentration data for multiplexing calculations, and these two laborious and tedious steps have become rate-limiting factors among the whole library preparation workflow. Here, with the aim of speeding up pre-sequencing sample preparation, we have proposed and validated the AIO-seq method, a novel method with critical improvements for library size selection and quantification. In AIO-seq, the target region concentration (TRC) of each library was calculated based on its size distribution pattern and total concentration, then multiple libraries were pooled into a single tube according to their TRC and expected data yield, after which the pooled single library was subjected to size selection and qualification for the target fragment, followed by quality control, and sequencing. Our AIO-seq method has simplified the entire library preparation process by replacing the laborious and tedious size selection and qualification steps with the all-in-one strategy that dramatically improves the efficiency, especially for large sample cohorts. We have shown here that the AIO-seq worked well for WGS and RNA-seq libraries, and have envisaged that it could be applied to other libraries determined from second-generation sequencing-based methods, like ChIP-seq, ATAC-seq, RAD-seq, etc. Library preparation for genetic mapping populations, like RIL populations, which are tolerant of inhomogeneous amounts of sequence data, was further simplified from the AIO-seq method by mixing the libraries directly after PCR. Using a maize BC_1_F_4_ population containing 116 individuals as a test, we constructed a genetic map and mapped the QTLs controlling plant height, ear height, and leaf angle successfully, using the simplified AIO-seq method.

## Results

### The mechanism of the AIO-seq workflow

The AIO-seq method was proposed based on three features of the NGS library which are critical but could easily be neglected. First, when analyzing the DNA size distribution pattern of the NGS library, the size-selected target DNA for the final sequencing was in a certain range of the whole library (Additional file 1: Figure S1), and a proportion of the target region was stable, and could be easily assayed by the Agilent 2100 Bioanalyzer, Qsep100™ or Fragment Analyzer™. We considered that the concentration of the target region, which would be size-selected for quantification and sequencing, could be determined by multiplying the original concentration of the whole library by the proportion of the target region. Then multiple libraries could be pooled according to the concentrations of their target regions and the expected yields of sequence data, if this region could be selected accurately. Second, among the tools available for size selection, the apparatus from Sage Science^®^ Incorporation could be used to recover the fragments of any target region from the whole library accurately and efficiently, which was ideal for the AIO-seq method. Third, though nearly a microgram of DNA was required for the size-selection after PCR amplification, only approximately 1–5 ng of DNA was loaded onto flowcells for cluster generation in each lane of the Illumina sequencer, thus the amount of DNA finally input into the sequencer for each library was low. For example, if 40 libraries were multiplexed to be sequenced in one lane, only ~ 0.2 ng of DNA was needed for each library. The DNA size-selected by the Sage tools could thus easily recover enough DNA for sequencing.

**Fig. 1:**
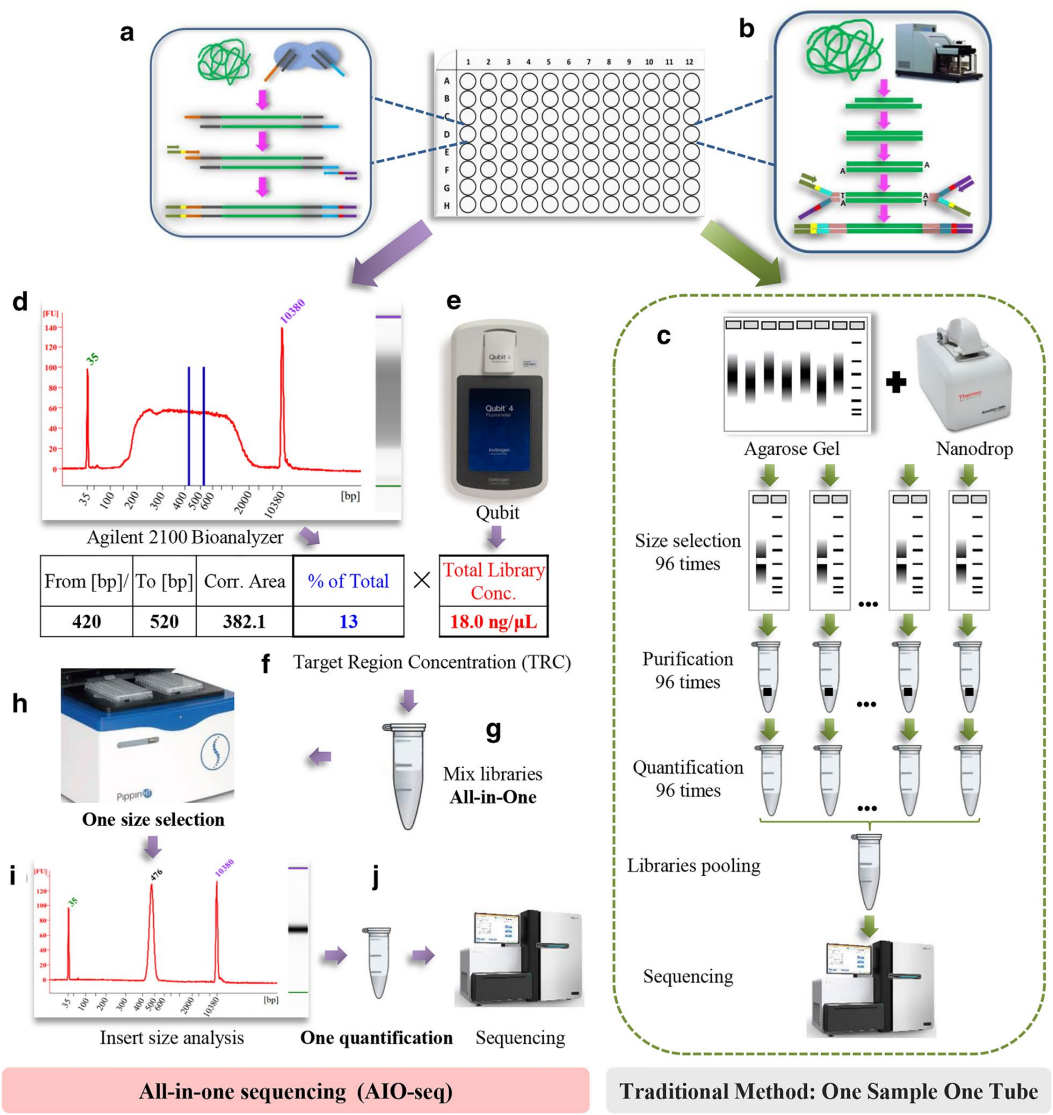
Flowchart for the All-in-One sequencing (AIO-seq) method. **a** The libraries were prepared using Tn5 transposase. **b** The process of mechanical fragmentation was used to prepare the libraries. **c** In the traditional protocol, the size selection, and quantification were processed using a one sample one tube method. **d** With the AIO-seq method, the library analyzed by the Agilent 2100 Bioanalyzer will give the fragment distribution pattern and the ratio of the target region (between the two blue lines) to the total library. **e** The concentration of the total library could be obtained by Qubit™ 4.0 Fluorometer. **f** The target region concentrations (TRC) were calculated within each library by multiplying the proportion of the target region from (**d**) and the total library concentration from (**e**). **g** Mixing the libraries in one tube according to the calculated TRC and their expected yields of the sequence data. **h**–**i** One size selection by Sage HT. **j** Quantification of the selected fragment by qPCR and sequencing

Based on the above-mentioned features, we designed the AIO-seq method with an improved size-selection and quantification strategy for high throughput and cost-effective NGS pre-sequencing library preparation (Fig. 1). The AIO-seq method was composed of the following steps. Initially the libraries were prepared using the Tn5 transposase (Fig. 1a), or with the process of DNA fragmentation, end-polishing, adaptors ligation (Fig. 1b), after PCR amplification and purification, in contrast with the traditional protocol where size selection and quantification occurred using the one sample one tube method (Fig. 1c), the fragment distribution pattern as well as the proportion of the target DNA size to the whole library were analyzed by the Agilent 2100 Bioanalyzer (Fig. 1d, Table 1), and the concentration of the whole library was assayed by Qubit™ 4.0 Fluorometer (Fig. 1e, Table 1). After that, the TRC of each library was calculated via the library concentration multiplied by the proportion of the target region (Fig. 1f, Table 1). Then the libraries could be multiplexed according to the calculated TRC and their expected data yields (Fig. 1g, Table 1). Finally, the libraries that were mixed in one tube, were subjected to a single fragment selection using the apparatus from Sage Science^®^ (Fig. 1h, i) and then a single quantification with qPCR (Fig. 1j). Our AIO-seq method condensed the most labor-intensive and low throughput steps of the library size selection and quantification process, from a one sample one tube method to an All-in-One method, and thus improved the preparation efficiency without impairing the quality of the library.

**Table 1:** Library pooling of 14 rice samples using AIO-seq

Group No.	Sample ID	Con. of lib (ng/μL)^a^	Ratio of 420–520 bp (%)^b^	Target region concentration (TRC, ng/μL)	Proportion of expected data yield in group^c^	Mass of mixed target region (ng)^c^	Vol. for mixing (μL)^d^	Final data yield (Gb)
A	1	21.00	11	2.31	1/7	20.0	8.66	6.38
	2	25.60	13	3.33	1/7	20.0	6.01	6.02
	3	19.60	13	2.55	1/7	20.0	7.85	7.20
	4	20.40	12	2.45	1/7	20.0	8.17	6.97
	5	24.80	13	3.22	1/7	20.0	6.20	7.03
	6	22.20	12	2.66	1/7	20.0	7.51	6.50
	7	24.20	12	2.90	1/7	20.0	6.89	6.74
B	1	20.20	10	2.02	1/7	20.0	9.90	5.79
	2	25.80	13	3.35	1/7	20.0	5.96	5.88
	3	22.00	12	2.64	1/7	20.0	7.58	6.44
	4	24.60	12	2.95	1/7	20.0	6.78	5.95
	5	21.40	13	2.78	1/7	20.0	7.19	5.46
	6	24.60	13	3.20	1/7	20.0	6.25	5.96
	7	26.20	13	3.41	1/7	20.0	5.87	5.74

^a^Con. of lib means the initial concentration of library for each individual assayed by Qubit™ 4.0 Fluorometer

^b^Analyzed by Agilent 2100 Bioanalyzer, indicates the ratio of the fragment between 420 and 520 bp, which will be size selected for sequencing

^c^For each group, seven samples were processed together and each sample was expected to have an equal yield of data, thus the proportion of expected data yielded for each sample was 1/7, and we mixed equal amounts of the target regions (20.0 ng) for each

^d^Vol. for mixing represents the volume of each library that needed to be pooled. Both the “Mass of mixed target region” and “Vol. for mixing” could be proportionately scaled up or down

### Proof-of-concept of AIO-seq demonstrated with 14 rice WGS libraries

**Fig. 2:**
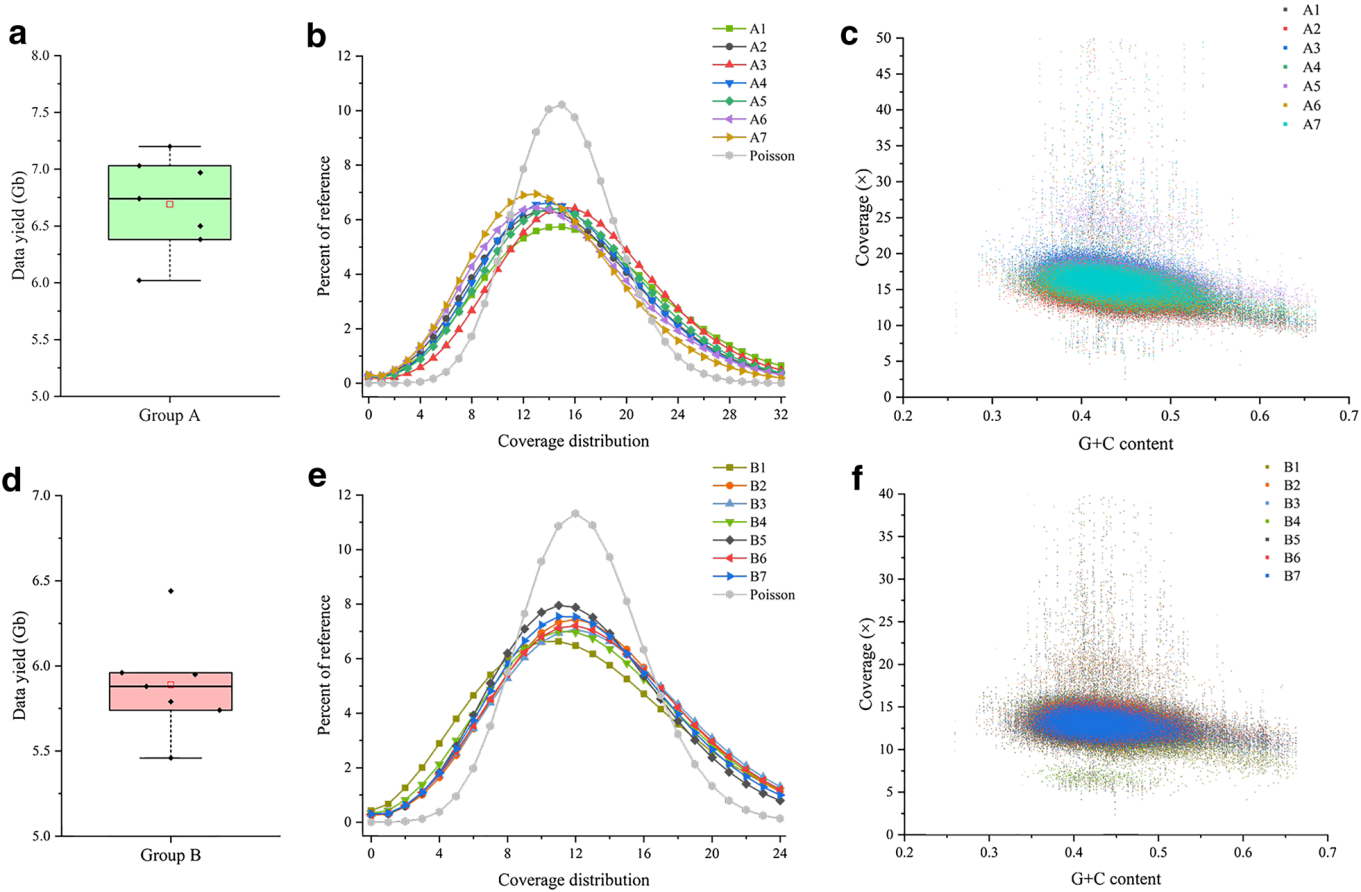
Data yield distribution and sequence coverage. Distribution of the data yields among the seven samples in group A (**a**) and the seven samples in group B (**d**). The coverage distribution as a percent of the Nipponbare reference genome among the samples in groups A (**b**) and B (**e**). The Poisson distribution which is expected if there were no bias is also shown with λ equal to the expected coverage among the genome. Coverage with respect to the G + C content of the reference in the 10 kb bins along the whole genome among samples in groups A (**c**) and B (**f**). For (**a**) and (**d**), the horizontal line and small open rectangle within the box plot indicate median and mean value, respectively. The extension of vertical lines indicates minimum and maximum observations excluding outliers. The black diamonds stand for the observations

As a proof of principle, we first tested the feasibility of the AIO-seq method with Tn5 transposase in 14 rice DNA samples. In this pilot test, the 14 samples were evenly divided into two groups (Group A and B), and then these two groups were subjected to library preparation separately using the AIO-seq method, where equal amounts of the target regions from each library were pooled with an expectation of even data yield outputs in each group (Table 1). After sequencing, a total of 46.8 and 41.2 gigabases (Gb) of raw data were generated from the two groups, respectively. As anticipated, the samples within each group had almost equal data yields (Fig. 2a, d) after demultiplexing, with 6.7 ± 0.39 Gb (mean ± SD) in group A and 5.9 ± 0.27 Gb in group B; and the coefficient of variation (CV) was 5.8% for group A and 4.7% for group B (Additional file 2: Table S1). Furthermore, for the seven samples in group A, the data outputs ranged from 6.0 to 7.2 Gb, of which six (85.7%) had a relative error (RE) of less than 10.0% (equal to absolute deviation (AD) of 0.67 Gb), and none of the samples had a RE above 25.0% (equal to AD of 1.7 Gb). Among group B, the data outputs ranged from 5.5 to 6.4 Gb and all samples (100%) had a RE of less than 10.0% (equal to AD of 0.59 Gb).

To further assess the data quality generated by the AIO-seq, the data were analyzed after mapping the clean reads of the samples to the Nipponbare reference genome (version 7) [18]. First, the mapping rates were above 97.77% and 97.79% in groups A and B, respectively (Additional file 2: Table S1), which were comparable with the libraries prepared according to the traditional protocol [1, 19, 20]. Second, relatively uniform coverage distributions, as a percent of the Nipponbare reference genome, were observed among the samples in both groups A and B (Fig. 2b, e). Furthermore, comparable biases in the coverage of the different G + C content bins (10 kb) of the reference genome were also found among the samples in both groups (Fig. 2c, f), with low representation at the two extremes.

Collectively, these results demonstrated that the WGS library prepared with the AIO-seq method, could produce evenly distributed data outputs among multiple samples, without using the one sample one tube method for size-selection and quantification, or impairing the sequence quality for further analysis.

### Preparation of multiple libraries for a whole lane of Hiseq X using AIO-seq

With the rapid increase in the sequencing capabilities of Illumina^®^ Hiseq X Series and NovaSeq Systems, the high yield from a single sequencing lane/run requires that more samples are multiplexed and sequenced simultaneously. Currently, the output per run could reach up to 1.8 terabases (Tb) and 6.0 Tb of sequence data from the Hiseq X and NovaSeq6000 Systems, respectively, while only several gigabases or less, for each sample, are required in most projects. To meet the technical demand, we tested the AIO-seq method with multiple samples mixed together in one tube, and only a single size selection and quantification step, followed by sequencing in a whole lane of the Hiseq X system, to get equal DNA sequence data for each sample.

**Fig. 3:**
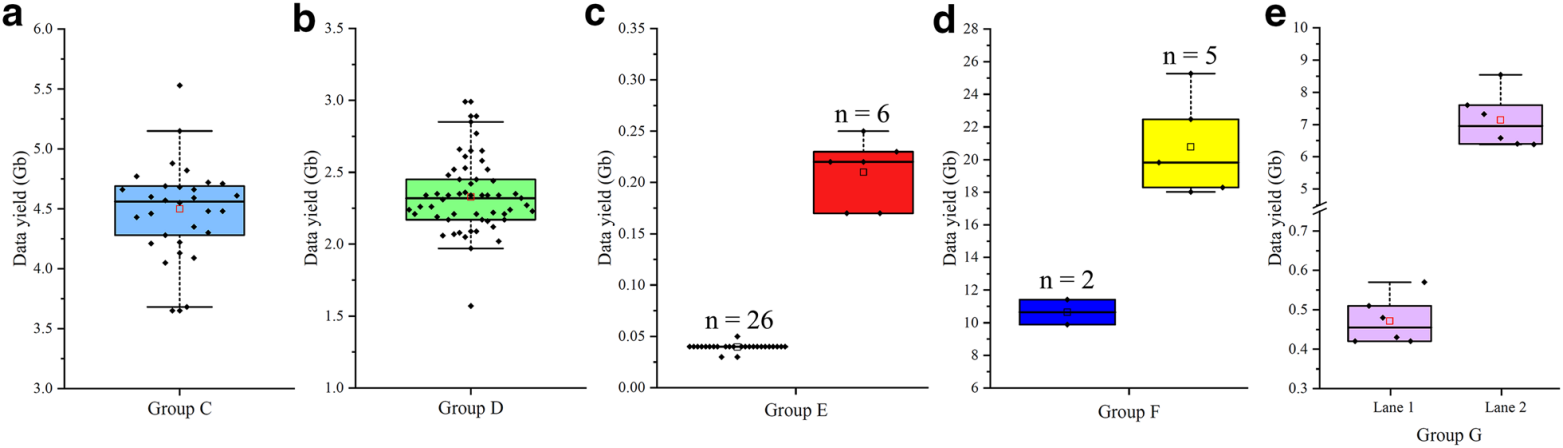
The distribution of data yields among the mixed samples in different groups. **a**, **b** Group C includes 30 and Group D includes 55 genomic DNA libraries sequenced in a single lane of a flowcell, separately. **c** In group E, a mixed library including 32 samples. Even data yields were expected among the 26 rice libraries and 6 maize libraries, respectively; and the data of each maize was expected to be approximately 5 × that of each rice sample. **d** In group F, a mixed library comprising seven tea libraries, two and five samples were expected to have even data yields, respectively; and the data output of each sample in the group of five was expected to be twice as much of each sample from the group of two. **e** Group G includes 6 RNA-seq libraries sequenced twice in two lanes. There are expectations of uneven data yields among samples within a mixed library. The horizontal line and small open rectangle within the box plot indicate median and mean value, respectively. The extension of vertical lines indicates minimum and maximum observations excluding outliers. The black diamonds stand for the observations

In this test, WGS libraries of 30 (Group C) and 55 (Group D) rice samples were prepared and sequenced in two Illumina^®^ Hiseq X lanes according to the AIO-seq method (Additional file 3: Table S2; Additional file 4: Table S3), and aimed to generate equal DNA sequence data for each individual in each group. Sequencing the two lanes generated 135.0 (Additional file 3: Table S2) and 128.1 Gb (Additional file 4: Table S3) of sequence data, respectively, and thus each sample should have 4.5 (135.0/30) and 2.3 (128.1/55) Gb of sequence data on average. After demultiplexing, an approximately equal data yield distribution was observed among the individuals in each group, with 4.5 ± 0.38 Gb in group C (Fig. 3a) and 2.3 ± 0.25 Gb in group D (Fig. 3b), and the CV was 8.4% in group C and 10.6% in group D. In group C, the maximum and minimum data outputs were 5.5 and 3.7 Gb, respectively, and 26 samples (86.7%) had a RE of less than 10.0% (equal to AD of 0.45 Gb), and only 2 samples (6.7%) had a RE above 25.0% (equal to AD of 1.1 Gb). In group D, the maximum and minimum data outputs were 3.0 and 1.6 Gb, respectively, and 37 samples (67.3%) had a RE below 10.0% (equal to AD of 0.23 Gb), and only 2 samples (3.6%) had a RE of more than 25.0% (equal to AD of 0.58 Gb). Taken together, these results suggest that the AIO-seq method could be utilized with more samples (30 and 55 in groups C and D, respectively), and still, only one size selection and quantification step was required for each group, to produce a comparably unbiased data distribution.

### AIO-seq can obtain different yields for each library within a single mixed library

When processing multiple projects, each library often requires different yields of sequence data. Traditionally, libraries were size-selected and quantified using the one sample one tube method, and then mixed according to their concentrations and the expected yields of the sequence data. Theoretically, the AIO-seq method could also pool all libraries, according to any expected data yield, by calculating the TRC for each sample. To validate this, two tests (Group E and Group F) were used to explore the ability of the AIO-seq method to get uneven sequence data from multiple samples, with a single size-selection and quantification step. For each group, the libraries were prepared and mixed according to the AIO-seq method (Additional file 5: Table S4; Additional file 6: Table S5).

After demultiplexing, most of the resulting data output for each sample within groups E and F coordinated with their predictions. Group E was comprised of 32 samples, 6 maize and 26 rice (Additional file 5: Table S4), and the maize and rice each had relatively even data distributions, with 0.21 ± 0.03 Gb and 0.04 ± 0.004 Gb, respectively (Fig. 3c). Furthermore, the CV was about 13.9% for the maize and 9.5% for the rice. Among the 6 maize samples, the maximum and minimum data outputs were 0.17 and 0.25 Gb, respectively, and 3 samples (50.0%) had a RE of less than 10.0% (equal to AD of 0.02 Gb), and no samples had a RE above 25.0% (equal to AD of 0.05 Gb). Among the 26 rice samples, the maximum and minimum data outputs were 0.032 and 0.046 Gb, respectively, and 20 samples (76.9%) had a RE of less than 10.0% (equal to AD of 0.004 Gb), and no samples had a RE above 25.0% (equal to AD of 0.01 Gb). More importantly, when the Chi square test was conducted on the data yields between the maize and rice pairwise, 153 of the 156 pairs (98.1%) had P > 0.05 and were found to be in accordance with the expected 5:1 ratio (Additional file 7: Table S6).

In group F, which included seven tea samples sequenced in a single lane of Hiseq X Ten (Additional file 6: Table S5), two samples generated nearly equal data outputs (9.9 Gb and 11.4 Gb), and the remaining five had approximately equal data yields (20.8 ± 2.7 Gb) (Fig. 3d). The CVs were 7.1% and 13.2% for the first two samples and the remaining five, respectively. Among the latter five samples, the maximum and minimum data outputs were 18.0 and 25.3 Gb, respectively, of which two samples (40.0%) had a RE of less than 10.0% (equal to AD of 2.1 Gb), and no samples had a RE above 25.0% (equal to AD of 5.2 Gb). Furthermore, the results of the Chi square test between the pairwise data yields of both the first two and each of the remaining five complied well (100%) with the expected 1:2 ratio, and all had P > 0.05 (Additional file 8: Table S7). These two successful tests both by a single size-selection and quantification strongly evidenced the flexibility, scalability, and robustness of the AIO-seq method.

### The AIO-seq strategy also worked well with RNA-seq libraries

In addition to the most popular WGS libraries, there are libraries from other sequencing methods based on second-generation sequencing platforms, for instance RAD-seq, RNA-seq, and ChIP-seq. However, regardless of the methods used, they all contain similar steps prior to sequencing, where size-selection and quantification are essential; hence we speculated that the AIO-seq method could also work for other types of NGS sequence libraries when sequenced with the Illumina^®^ platform. Thus, the performance of the AIO-seq for the RNA-seq library was further assessed, as it is a commonly used NGS method.

In this assay, six rice RNA-seq libraries (Group G) were pooled according to the AIO-seq method, as stated in the WGS library preparation, and equal yields of sequence data were expected (Additional file 9: Table S8). The pooled library was then subjected to a single size selection for the target region between 300 and 600 bp, and a single quantification by qPCR. After sequencing twice on two separate Hiseq X runs, a total of 2.8 Gb and 42.8 Gb of sequence data (Additional file 9: Table S8) was generated, respectively. As expected, comparable yields of sequence data were achieved among the pooled libraries sequenced on each lane with 0.47 ± 0.06 Gb and 7.1 ± 0.8 Gb (Fig. 3e), and with CVs of 11.7% and 10.9%, respectively. In more detail, three samples (50.0%) had a RE of less than 10.0% (equal to AD of 0.05 Gb), and no sample had a RE of more than 25.0% (equal to AD of 0.12 Gb) in the first run (lane 1), and three samples (50.0%) had a RE of less than 10.0% (equal to AD of 0.71 Gb), and no sample had a RE higher than 25.0% (equal to AD of 1.8 Gb) in the second run (lane 2). Hence, we could draw the conclusion that the AIO-seq method described, worked well with RNA-seq libraries.

### QTL mapping in a maize BC_1_F_4_ population with simplified AIO-seq

QTL mapping that utilizes WGS data has been widely used in crops. Two bioinformatics pipelines were developed for rice QTL mapping using RILs with only 0.02 × coverage of the WGS data for each line [21, 22]. Hence, we speculated that for population analysis with RIL, mixing the final PCR products directly without calculating their TRC in the AIO-seq method, would increase the deviation of the final yields for each sample, but as far as 0.02 × coverage might be acceptable, a few samples with extreme deviation should not affect the QTL mapping. Hence, we further simplified the AIO-seq method by mixing the PCR products directly without assaying the concentrations and TRC for a rice RIL population with 109 lines and mapped the QTLs successfully (Chang, et al., Unpublished data).

We also applied the simplified AIO-seq method in a maize BC_1_F_4_ population to test its performance in crops with more complex genomes. For this maize BC_1_F_4_ population with 116 lines, two fragment sizes were selected by Sage ELF with peaks of 465 bp (Library 1) and 516 bp (Library 2), and they were sequenced in different lanes by Hiseq X. A total of 180.7 Gb data (~ 0.74 × genome coverage for each sample; Additional file 10: Table S9) were generated after merging the raw reads generated from Library 1 with the total bases of 126.6 Gb (1.1 ± 0.33 Gb, a full lane) and 54.1 Gb (0.47 ± 0.14 Gb) from Library 2 (Additional file 11: Figure S2), respectively. In brief, there were 66 samples (56.9%) that had a RE of less than 10% (equal to 0.1 Gb) and 15 samples (12.9%) that had a RE above 25% (equal to 0.25 Gb) for Library 1, whose CV was 30.3%. Library 2 had a CV of 29.9%, and 58 samples (50.0%) were found to have a RE of less than 10% (equal to 0.05 Gb) and 17 samples (14.7%) had a RE above 25% (equal to 0.12 Gb). These results indicated that the population sequenced using the simplified AIO-seq method could achieve an acceptably even data output among individuals.

**Fig. 4:**
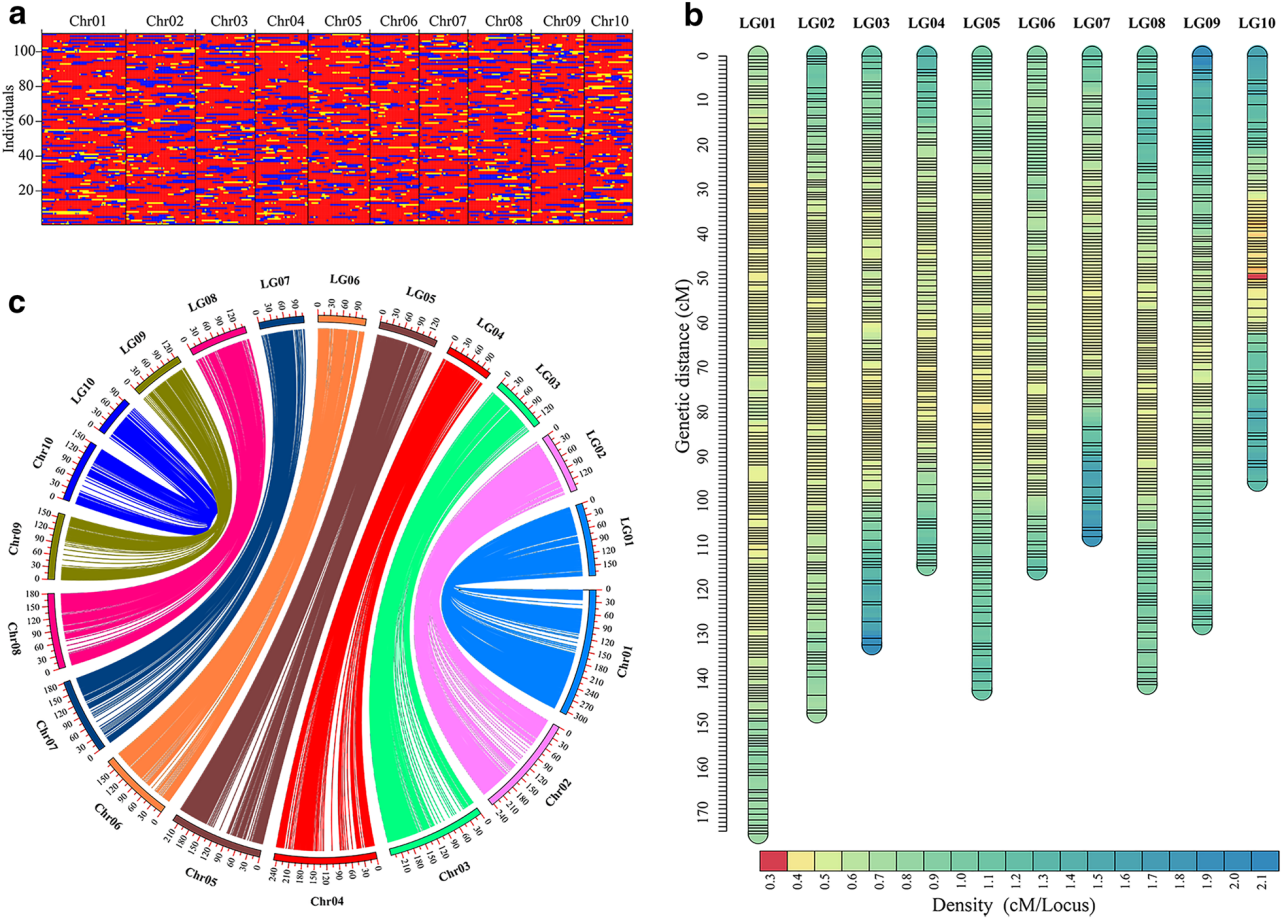
Map construction and collinearity analysis of a maize BC_1_F_4_ population. **a** Recombination bin map of the 110 maize BC_1_F_4_ lines from a cross between CML486 and Lx9801. Red: Lx9801 genotype; blue: CML486 genotype; yellow: heterozygote. **b** A high-density genetic map constructed with 2264 bin markers from AIO-seq. **c** Collinearity analysis between the genetic map and the physical map of B73 reference genome (version 4). The corresponding relationship and the position relationship between the maize chromosomes (Chr) and the linkage groups (LG) of the genetic map are shown

Using the data generated by the above simplified AIO-seq method, we subsequently conducted plant height, ear height, and leaf angle (Additional file 12: Figure S3) related QTL mapping analysis with the maize BC_1_F_4_ population. Among the three traits, leaf angle had the biggest heritability, followed by plant height, and ear height (Additional file 13: Table S10). After SNP calling and filtering, about 2,287,037 high-quality SNPs or 1.1 SNPs/kb were obtained between the two parents, with ~ 14 × genome coverage for both (Additional file 14: Table S11), and on average 398,313 SNPs (~ 188.8 SNPs/Mb) were detected for each sample. Next, a sliding-window based method was used to identify the recombination breakpoints along the chromosomes in each individual, and a total of 3780 recombination breakpoints were identified from the 110 BC_1_F_4_ lines (6 highly heterozygous lines were excluded), with an average of 34.4 per sample (Fig. 4a). After that, a high-density genetic map was constructed with 2264 bin markers (Fig. 4b, Additional file 15: Table S12), ranging from 100.2 kb to 38.5 Mb, which also exhibited a high collinearity (*r* = 0.93) with the B73 reference genome (version 4) (Fig. 4c). At last, a total of 19 QTLs were detected for plant height, ear height, and leaf angle, with physical regions that spanned from 0.8 Mb to 55.4 Mb (Table 2), among which three stable QTLs (*qLA1a*, *qLA2a,* and *qLA2b*) that control leaf angle, could be detected over the 2 years consistently. Taken together, the results showed that simplified AIO-seq could be applied to QTL mapping efficiently for crops, not only with simple genomes like rice, but also those with complex genomes like maize.

**Table 2:** QTLs identified for plant height, ear height and leaf angle using a high-density genetic map

Trait^a^	Year	Chromosome	QTL	Location (cM)	Region (Mb)	LOD^b^	R^2^ (%)^c^	Addictive effect^d^
PH	2016	3	*qPH3a*	36.0–44.9	200.8–210.7	3.4	10.0	5.1
	2016	3	*qPH3b*	95.4–100.8	6.9–9.7	3.5	12.3	− 5.8
	2017	4	*qPH4*	74.6–81.8	23.0–78.4	3.3	9.9	5.2
	2017	6	*qPH6a*	81.6–87.7	37.8–91.6	2.8	8.3	− 5.1
	2017	6	*qPH6b*	87.7–93.2	15.1–37.8	4.0	12.4	− 6.2
	2016	8	*qPH8a*	106.4–110.6	12.7–13.5	3.7	11.2	− 6.4
	2017	8	*qPH8b*	123.4–139.8	1.4–5.1	2.6	7.5	− 4.3
EH	2017	3	*qEH3*	46.3–56.4	183.2–199.6	3.2	9.3	− 3.7
	2016	4	*qEH4*	33.1–42.3	184.8–200.8	3.2	10.3	− 4.5
	2017	5	*qEH5*	21.0–28.7	210.5–215.6	3.6	10.4	− 4.4
	2017	10	*qEH10*	88.2–94.3	148.6–149.8	4.8	15.4	− 5.1
LA	2016, 2017	1	*qLA1a*	120.3–132.3	24.4–68.6	3.0–10.6	6.7–26.8	− (2.4–4.1)
	2016	1	*qLA1b*	133.6–135.0	19.3–24.4	5.7	14.9	− 3
	2016	1	*qLA1c*	141.5–149.3	11.6–15.2	3.1	6.9	− 2.3
	2016, 2017	2	*qLA2a*	120.0–122.4	11.3–13.0	3.6–4.7	8.7–11.7	− (2.3–2.7)
	2016, 2017	2	*qLA2b*	128.0–143.4	1.9–9.1	4.6–8.4	12.4–20.8	− (2.7–3.6)
	2016	3	*qLA3*	88.2–90.7	12.0–20.6	5.4	11.0	− 2.6
	2016	7	*qLA7a*	44.2–49.0	131.2–142.0	3.9	8.1	2.4
	2016	7	*qLA7b*	52.6–59.8	110.8–126.7	3.7	7.9	2.2

^a^Trait is the name of the component of the plant architecture: PH for plant height, EH for ear height, LA for leaf angle

^b^LOD means logarithm of odds

^c^R^2^ indicated the percentage of phenotypic variation explained by QTL

^d^Positive or negative addictive effect value indicates that the allele from Lx9801 or CML486 increases the phenotypic value, respectively

## Discussion

In this study, we have developed and presented the AIO-seq method, which is a highly efficient and cost-effective improvement for the preparation of NGS libraries. It replaces of the standard ‘one sample, one tube’ method for the size selection and quantification step, with a multiple samples ‘all-in-one tube’ method. We have demonstrated the practicability of our AIO-seq method for multiplexing WGS libraries, where the mixed samples in a single tube had either the same or different expected data yields, to sequence in a whole or partial Hiseq X lane. Furthermore, AIO-seq also worked for an RNA-seq library and has the potential to be applied to numerous other NGS libraries. Moreover, the further simplified AIO-seq method could be applied to RIL populations for QTL mapping in both rice and maize.

Since the first report of the application of Tn5 transposase in shotgun library preparation [9], several novel genome research methods have been developed, based on the straightforward tool for DNA fragmentation and adaptor-ligation in library preparation. For example, using methylated adapters, transposase was exploited in whole-genome bisulfite sequencing research [23]. The Tn5 transposase enzyme can stay bound to its DNA substrate and thus maintains the contiguity of the target DNA after transposition, using this feature, the Contiguity-Preserving Transposition sequencing (CPT-seq) and single-tube Long Fragment Read (stLFR) were developed for whole genome de novo assembly and haplotype sequencing [24, 25]. Tn5 transposase was also used to detect genome structures and chromatin accessibility by ATAC-seq, transposase-mediated analysis of chromatin looping (Trac-looping) and Cleavage Under Targets and Tagmentation (CUT&Tag) method [26–28]. Since extremely low levels of DNA were required for Tn5 transposase tagmentation and adaptor ligation, it was also widely used in DNA/RNA-seq with low material input, even with single cells [29, 30].

With the application of Tn5 transposase in whole genome sequencing, researchers have developed a variety of approaches to increase the throughput and decrease the cost for library preparation, especially for large cohorts of samples. For example, for megabase-sized genomes, the costs of library preparation could be decreased sixfold by carrying out the tagmentation reaction in volumes as small as 2.5 μL, and replacing the costly reagents with cheaper equivalents, as well as omitting unnecessary steps [31, 32], though this protocol was originally developed for small microbial genomes (< 15 Mb), it has been validated to work well for the teleost fish with a genome size of ~ 730 Mb [33]. However, these methods still processed samples using a one tube one sample method, and the compensation of low DNA inputs in small tagmentation reaction volumes increased the PCR cycle numbers from 5 to 13, resulting in uneven genome coverage. In our AIO-seq method, we have used half of the recommended DNA input and reaction volumes and maintained a PCR cycle number of 5. With this method, approximately 200 ng of PCR product could be obtained for each sample. With the traditional method, this amount was not enough for the following size selection, however, instead of processing the samples with the one sample one tube method, we pooled dozens of samples together (Fig. 3a–c) for size selection according to their TRC and expected yield, since less than 1 ng DNA for each sample was required for loading onto the flowcell. Thus, our method simplified the steps (Fig. 1), but did not affect the library quality (Fig. 2b, c, e, f).

While our method simplified the whole library preparation process dramatically, requiring less labor and reagents for large-scale experiments, the cost of the transposase kit was still a limiting factor. Fortunately, Picelli et al. [10] introduced a simple and robust procedure for Tn5 transposase production, and demonstrated that the performance of their home-made enzyme equaled that of the commercial one. Their enzyme purification was further improved using an N-terminal His_6_-Sumo3 tag [34]. These improvements combined with our simplified AIO-seq will make the library preparation affordable for experiments with any number of samples, the low cost for whole genome genotyping will increase the application of genome selection in plant breeding research.

A couple of studies have validated that the QTL mapping resolution can be improved with larger population sizes and greater marker density [35–37], however, the cost for whole genome genotyping either by DNA array or NGS-based methods limited the population size for QTL mapping. In plant breeding, a greater number of samples are needed for whole genome genotyping at a low cost. Our AIO-seq method, especially the simplified AIO-seq, omitted all of the unnecessary steps for the tradeoff of some samples that had extreme but acceptable deviations in their data yields (Additional file 11: Figure S2), and could thus meet the requirements for QTL mapping and plant breeding projects with large sample population sizes. Compared with the traditional methods for WGS, the simplified AIO-seq not only increased the efficiency by approximately threefold, in terms of both the total and hands-on time required (Table 3), but also reduced the total cost by 2/3 or even more (Additional file 16: Table S13) when 96 samples were prepared. Using the data generated by the simplified AIO-seq method, the QTL mapping for three traits (plant height, ear height, and leaf angle) in this population were completed. Interestingly, the two major QTLs for the leaf angle, *qLA1a* and *qLA2b*, that were identified in both years, were positioned between 24.4 and 68.6 Mb on chromosome 1 and 1.9–9.1 Mb on chromosome 2, respectively, where *drl1* [38] and *lg1* [39] were located, that had previously been reported to be responsible for leaf angle. Another QTL, *qLA2a* was located between 11.3 and 13.0 Mb on chromosome 2, and was also detected in both years. These results illustrated the AIO-seq is a powerful tool for QTLs mapping at a low cost, and we envisaged that the AIO-seq described here, with its excellent robustness, scalability, low cost, and high throughput characteristics, could be widely applied in future research involving plant breeding, population genetics, and related projects.

**Table 3:** Comparison of the labor input between traditional method and simplified AIO-seq for 96 RIL samples

Step	Traditional pipeline^a^		Simplified AIO-seq pipeline	
	Time needed (day)	Hands-on time (hour)	Time needed (day)	Hands-on time (hour)
Library preparation^b^	1.5	8	0.5	5
Size profile analysis	0.5	2	0	0
Size selection	0.5	6	0.1	0.2
qPCR of library	0.2	5	0.1	0.2
Total	2.7	21	0.7	5.4

^a^Traditional pipeline is started from mechanical or enzymatic fragmentation

^b^Library preparation includes steps from genomic DNA to the finish of the PCR

## Conclusions

In this study, we developed an AIO-seq method, which could condense the size selection and quantification steps in NGS library preparation, from a ‘one sample one tube’ method, to a ‘multiple samples one tube’ method, and thus substantially reduce the overall time and labor required to prepare sequencing libraries. Moreover, as sequencing library preparations are limiting steps for large sample cohorts, our method has a great perspective on population genetic studies and plant breeding research.

## Methods

### The sources of plant materials

The 14 samples in groups A and B for the preliminary AIO-seq tests were rice EMS mutants developed in our lab using the variety Kongyu 131 (*Oryza sativa* ssp. *japonica*). The 111 samples (30 in group C, 55 in group D, 26 in group E) then used to test the amounts of data yield, were rice RIL populations derived from the cross of Huanghuazhan and Shuanggui 36 [40]; the remaining six samples in group E were maize (*Zea mays*) breeding materials from our ongoing breeding programs at Henan Agricultural University; the six tea (*Camellia sinensis*) accessions used in group F were from the Tea Research Institute of Chinese Academy of Agricultural Sciences (CAAS).

For the simplified AIO-seq tests in the maize RIL and QTL mapping, a mapping population consisting of 116 BC_1_F_4_ lines was developed by single-seed descent from the BC_1_ population: (CML486 × Lx9801) × Lx9801, for which CML486 is an elite tropical inbred line from CIMMYT, and Lx9801 is a dent inbred line from Tangsipingtou [41]. The parents and their progeny were grown in experimental fields in Changge city (N34*°*13′, E113*°*46′), Henan province, over two consecutive years (2016 and 2017), following the regular agriculture management practices during the growing seasons. A total of three agricultural traits were recorded for the parents and all BC_1_F_4_ lines. Plant height and ear height were measured using a similar method as previously described [42], and leaf angle was determined for four leaves above the primary ear as the angle of each leaf from a plane defined by the stalk below the node subtending the leaf [43]. Standard analysis of variance and broad-sense heritability calculations were performed using QTL IciMapping v4.1 software [44].

### AIO-seq library preparation

The genomic DNA (gDNA) from all the materials used in this study was extracted with CTAB [45], and RNA was isolated using TRIzol™ Reagents from Thermo Fisher (Cat. 15596026).

TruePrep^®^ DNA Library Prep Kit V2 for Illumina^®^ (Vazyme Biotech, Nanjing, China, Cat. TD501-02) or customized Tn5 transposase from TransGen Biotech (Beijing, China), was used for the AIO-seq library preparation. A detailed protocol can be found in the supplementary documentation (Additional file 17: Method S1). Briefly, based on the optimal ratio of input gDNA to transposase, 30 ng of gDNA in 25 μL reaction volumes was fragmented into 200–1000 bp fragments with broad peaks of smooth distribution curves, then 1 μL 2.6% SDS was added to strip the Tn5, after which the tagmented gDNA was subjected to 5–6 cycles of PCR amplification, followed by purification with 1.8 × VAHTS™ DNA Clean Beads (Vazyme Biotech, Nanjing, China, Cat. N411-02) and then eluted in 20 μL (or less) sterile ddH_2_O.

The mRNA was enriched from approximately 50 ng of high-quality total RNA using NEXTflex™ Poly(A) Beads (PerkinElmer, Texas, USA, Cat. NOVA-512979), then NEXTflex™ Rapid RNA-Seq Kit (PerkinElmer, Texas, USA, Cat. NOVA-5138-01) for the Illumina^®^ platform was used to prepare RNA-Seq libraries following the manufacturer’s protocols.

After library preparation, the Qubit™ dsDNA HS Assay Kit (Thermo Fisher Scientific, Cat. Q32854) was used to quantify the concentration of libraries, and the Agilent 2100 Bioanalyzer (Agilent Technology, CA, USA) was used to assay the size distribution and the proportion of target regions (420–520 bp for paired-end 150 bp sequencing) to the whole library. The TRC of each library was calculated by multiplying the proportion of the target region from the Agilent 2100 Bioanalyzer and the total library concentration from the Qubit™ 4.0 Fluorometer (Invitrogen, NY, USA), then mixing the libraries in one tube according to the calculated TRC and their expected data yields. For the RIL samples, the PCR products were mixed directly without the Agilent 2100 Bioanalyzer and Qubit assay, followed by purification with 1.8 × VAHTS™ DNA Clean Beads.

After library pooling, an automatic cassette-based SageELF (Sage Science, MA, USA) with a unique capacity to simultaneously isolate 12 different discrete size fractions from a single loaded sample was used for size selection to get a tight fragment span, running with the time-based mode following the manufacturer’s procedures. After size selection, an Agilent 2100 Bioanalyzer was employed to analyze the quality of the fractionated fragments and chose ones with well-constrained size ranges located in the target region for further high throughput sequencing. All libraries in this study were sequenced on an Illumina^®^ HiSeq X Ten system with paired-end 150 bp sequencing.

### Data quality control and genotype calling

After demultiplexing, clean reads were obtained according to the previously reported quality-filtering parameters [46] from the raw sequence data. Then high-quality reads were mapped to the Nipponbare reference genome (version 7) [18] using the Burrows-Wheeler Aligner v0.7.5 (BWA) [47]. After mapping, coverage distribution curves were generated by calculating the number of times that each base of the genome was sequenced and then plotting the frequency of each level of coverage, and coverage by G + C contents graphs were obtained by dividing the rice reference genome into 10 kb bins and then calculating the G + C content within each bin, followed by plotting the coverage of that bin, which was similar to a previous study [9].

For genotype calling of the maize BC_1_F_4_ population, the raw reads were trimmed by Trimmomatic v0.36 [48] and then mapped to the maize B73 reference genome (version 4) [49] using BWA. Only the reads uniquely mapped to the reference genome were used to call SNPs. PCR duplicates were removed by the Picard tools v1.119 (http://broadinstitute.github.io/picard/). SNP calling was performed using the SAMtools software v1.5 [50]. For two parental lines, SNPs were filtered using a custom Perl script with the following stringent criteria: (a) homozygous and polymorphic between parents; (b) the depth of the SNPs in each parental line ≥ 4 × ; (c) mapping quality (MQ) value ≥ 60; (d) phred-scaled quality (QUAL) value ≥ 60; (e) not located in the TE regions of the reference genome. Subsequently, genotype calling of each BC_1_F_4_ line was carried out based on the high-quality SNP alleles between parents. The candidate SNPs of each progeny should meet the following criteria: (a) MQ ≥ 60; (b) QUAL ≥ 60; (c) homozygous and consistent with either of the parental genotypes.

### Bin map construction and QTL analysis

A sliding window approach [21] was used to construct bin maps of BC_1_F_4_ lines. Briefly, first, after all SNPs of the BC_1_F_4_ lines were called, a Perl script, *Seq2Bin*, implemented in SEG-Map package [51] was applied for recombination breakpoint detection and recombination map construction. Then, to prevent the detection of false double-crossovers on the chromosome, the genotypes of the raw blocks between two recombination breakpoints in each sample were manually corrected to match with the adjacent genotype on condition that short heterozygous blocks (< 0.5 Mb) were located in the middle of the same continuous homozygous genotype blocks or short homozygous blocks (< 0.5 Mb) were located in the middle of the continuous heterozygous genotype blocks in the raw recombination map. Six samples with > 300 recombination breakpoints were excluded from further analysis. After that, the recombination maps of the remaining 110 BC_1_F_4_ lines were aligned and compared over 100 kb minimum intervals along each chromosome. The adjacent 100 kb intervals with the same genotype in the whole BC_1_F_4_ were considered as a recombination bin. Finally, the resultant bins served as genetic markers for linkage map construction using QTL IciMapping v4.1 software [44]. Collinearity between the genetic map and B73 reference genome (version 4) was determined by plotting genetic marker positions (in centimorgans) against their physical midpoint positions (in Mb) using TBtools v0.6673 [52].

For QTL analysis, composite interval mapping was performed with Windows QTL Cartographer v2.5 (https://brcwebportal.cos.ncsu.edu/qtlcart/WQTLCart.htm). The threshold score of the logarithm of odds (LOD) for each trait was determined by performing a 1000 permutation test with 5% probability. The location of a QTL was described according to its LOD peak position and the surrounding 2-LOD region calculated by WinQTLCart. QTLs controlling the same trait and commonly detected over 2 years with overlapped mapping regions were consolidated to one QTL as previously described [53].

## Supplementary information

**Additional file 1: Figure S1.** Size selection of target region from a whole library.

**Additional file 2: Table S1.** Data metrics from sequencing of 14 rice libraries prepared using AIO-seq method.

**Additional file 3: Table S2.** Library pooling and data metrics in group C with 30 rice libraries pooled.

**Additional file 4: Table S3.** Library pooling and data metrics in group D with 55 rice libraries pooled.

**Additional file 5: Table S4.** Library pooling and data metrics in group E with 32 libraries pooled.

**Additional file 6: Table S5.** Library pooling and data metrics in group F with seven tea libraries pooled.

**Additional file 7: Table S6.** Chi square test in group E with 32 libraries pooled.

**Additional file 8: Table S7.** Chi square test in group F with seven tea libraries pooled.

**Additional file 9: Table S8.** Library pooling and data metrics in group G with 6 rice RNA-seq libraries pooled.

**Additional file 10: Table S9.** Data metrics from sequencing of a maize BC_1_F_4_ population including 116 lines using a simplified AIO-seq method.

**Additional file 11: Figure S2.** Distribution of the data yield among 116 BC_1_F_4_ lines with a simplified AIO-seq method.

**Additional file 12: Figure S3.** Correlation coefficients analysis of three traits in a maize BC_1_F_4_ population across 2 years 2016 (a) and 2017 (b).

**Additional file 13: Table S10.** Descriptive statistics and analysis of variation for three traits in 2016 and 2017.

**Additional file 14: Table S11.** Data metrics of two parents in a maize BC_1_F_4_ population using a simplified AIO-seq method.

**Additional file 15: Table S12.** Characteristics of the high-density genetic linkage map.

**Additional file 16: Table S13.** Comparison of the estimated cost between traditional method and simplified AIO-seq for 96 samples.

**Additional file 17: Method S1.** Protocol for the AIO-seq method.

## Data Availability

The datasets used and/or analyzed during the current study are available from the corresponding authors on reasonable request.

## Abbreviations

AD: Absolute deviation
AIO-seq: All-in-one sequencing
ATAC-seq: Assays for transposase-accessible chromatin with high-throughput sequencing
BWA: Burrows-Wheeler Aligner
ChIP-seq: Chromatin immunoprecipitation coupled with massively parallel sequencing
CPT-seq: Contiguity-preserving transposition sequencing
CUT&Tag: Cleavage under targets and tagmentation
CV: Coefficient of variation
ddPCR: Droplet digital PCR
Gb: Gigabases
gDNA: Genomic DNA
LOD: Logarithm of odds
MQ: Mapping quality
NGS: Next generation sequencing
qPCR: Quantitative PCR
QUAL: Phred-scaled quality
RAD-seq: Restriction site associated DNA sequencing
RE: Relative error
RIL: Recombinant inbred lines
stLFR: Single-tube long fragment read
Tb: Terabases
TRC: Target region concentration
Trac-looping: Transposase-mediated analysis of chromatin looping
WGS: Whole genome sequencing
