# Supplementary material for: All-in-one sequencing: an improved library preparation method for cost-effective and high-throughput next-generation sequencing
Source: Plant Methods. 2020 May 24;16:74. doi: 10.1186/s13007-020-00615-3 (PMC7247233; doi:10.1186/s13007-020-00615-3)
Supplement: Supplementary file 1 — Additional file 1: Figure S1. Size selection of target region from a whole library. [file 13007_2020_615_MOESM1_ESM.pdf]

(a)

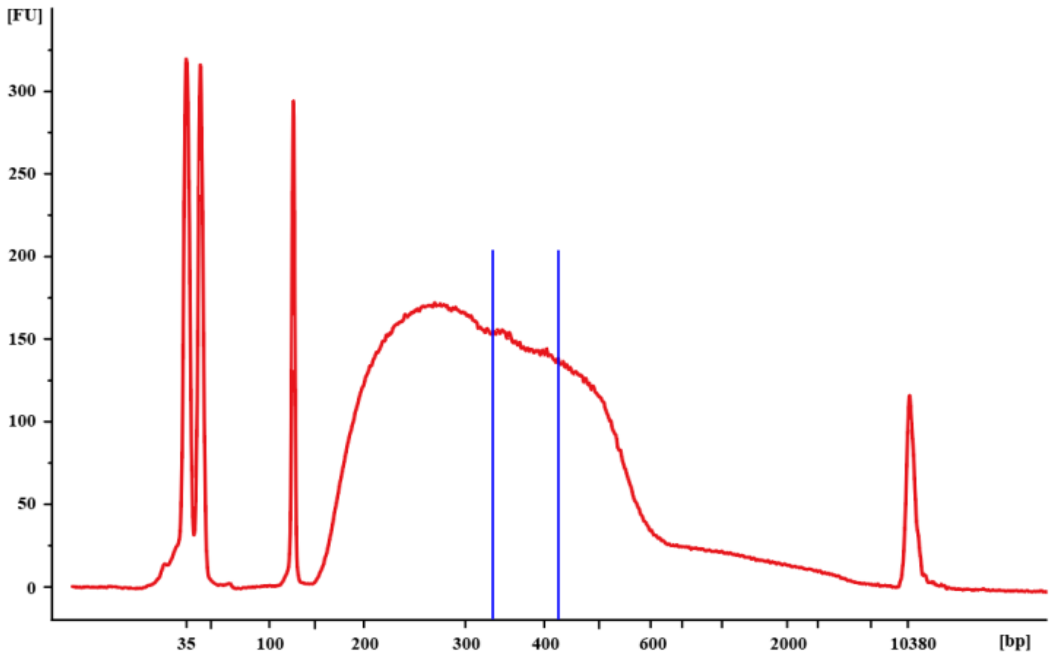

From [bp]/	To [bp]	Corr. Area	% of Total
340	440	1408.8	**19**

(b)

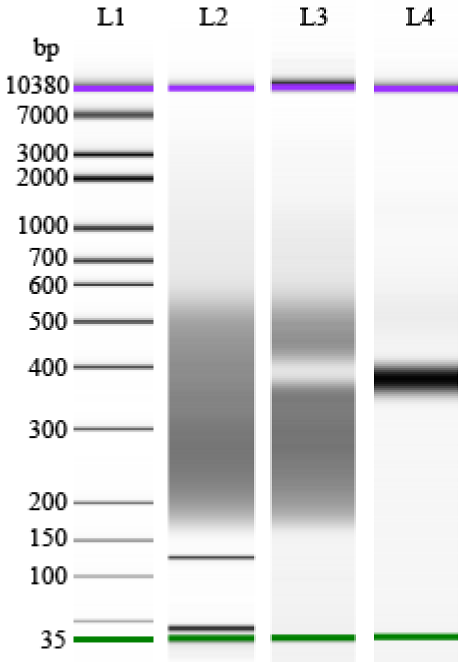

Figure S1. Size selection of target region from a whole library. (a) Size distribution of a library analyzed by Agilent 2100 Bioanalyzer where the blue line delimited the range of the region to be size selected for sequencing. The table below the figure showed the range of target region and its proportion to the whole library. (b) Gel image of target region selection using Pippin HT instrument. L1, DNA ladder; L2, The library before size selection; L3, The remaining fragment after target region selection; L4, fragment of selected target region.
