# Supplementary material for: All-in-one sequencing: an improved library preparation method for cost-effective and high-throughput next-generation sequencing
Source: Plant Methods. 2020 May 24;16:74. doi: 10.1186/s13007-020-00615-3 (PMC7247233; doi:10.1186/s13007-020-00615-3)
Supplement: Supplementary file 11 — Additional file 11: Figure S2. Distribution of the data yield among 116 BC1F4 lines with a simplified AIO-seq method. [file 13007_2020_615_MOESM11_ESM.pdf]

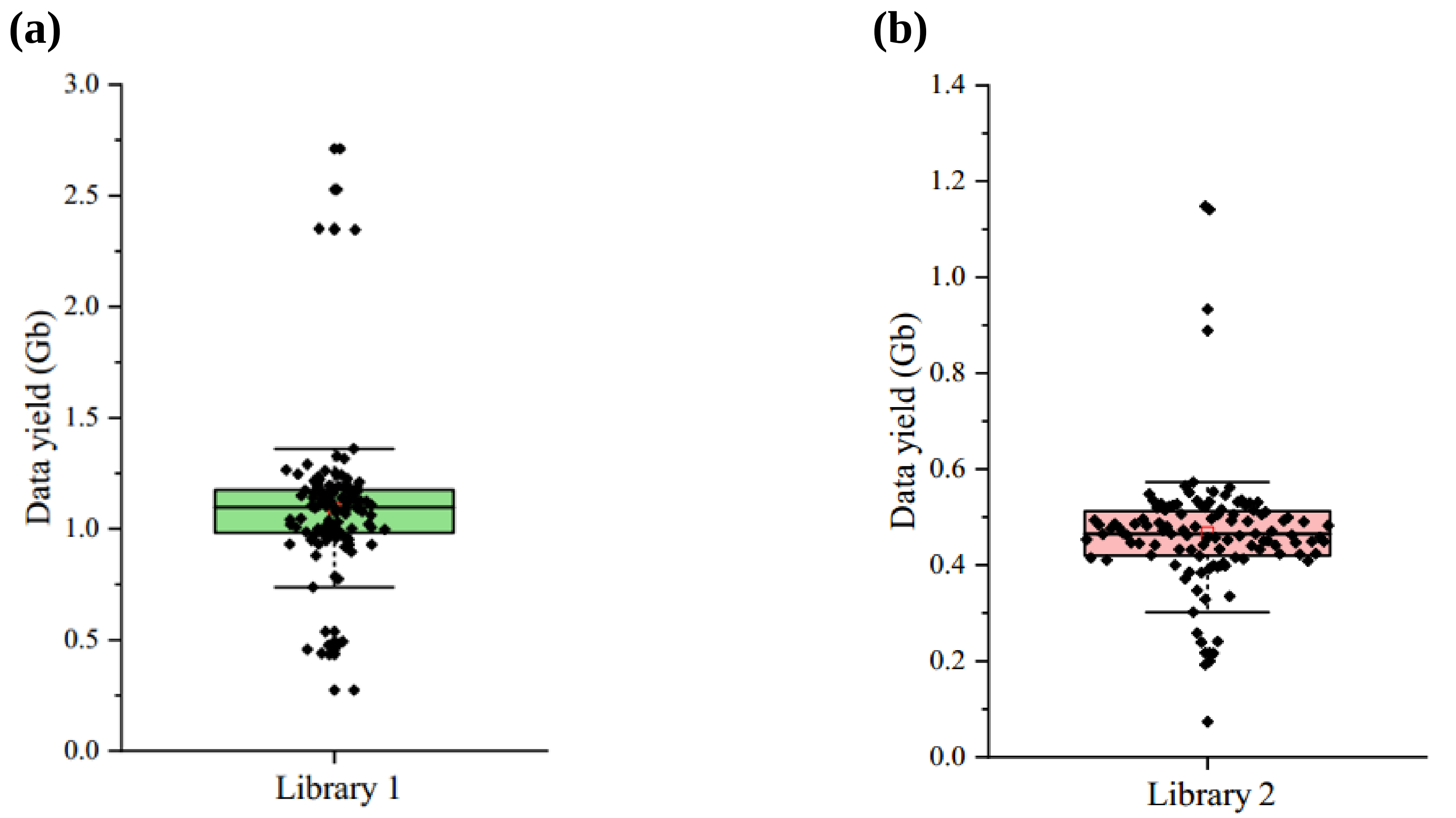

Figure S2. Distribution of the data yield among 116 BC_1F_4 lines with a simplified AIO-seq method. (a) Library 1 was a fragment with peak size of 465 bp. (b) Library 2 is a fragment with peak size of 516 bp. The horizontal line and small open rectangle within the box plot indicate median and mean value, respectively. The extension of vertical lines indicates minimum and maximum observations excluding outliers. The black diamonds stands for the observations.
