# Supplementary material for: All-in-one sequencing: an improved library preparation method for cost-effective and high-throughput next-generation sequencing
Source: Plant Methods. 2020 May 24;16:74. doi: 10.1186/s13007-020-00615-3 (PMC7247233; doi:10.1186/s13007-020-00615-3)
Supplement: Supplementary file 12 — Additional file 12: Figure S3. Correlation coefficients analysis of three traits in a maize BC1F4 population across 2 years 2016 (a) and 2017 (b). [file 13007_2020_615_MOESM12_ESM.pdf]

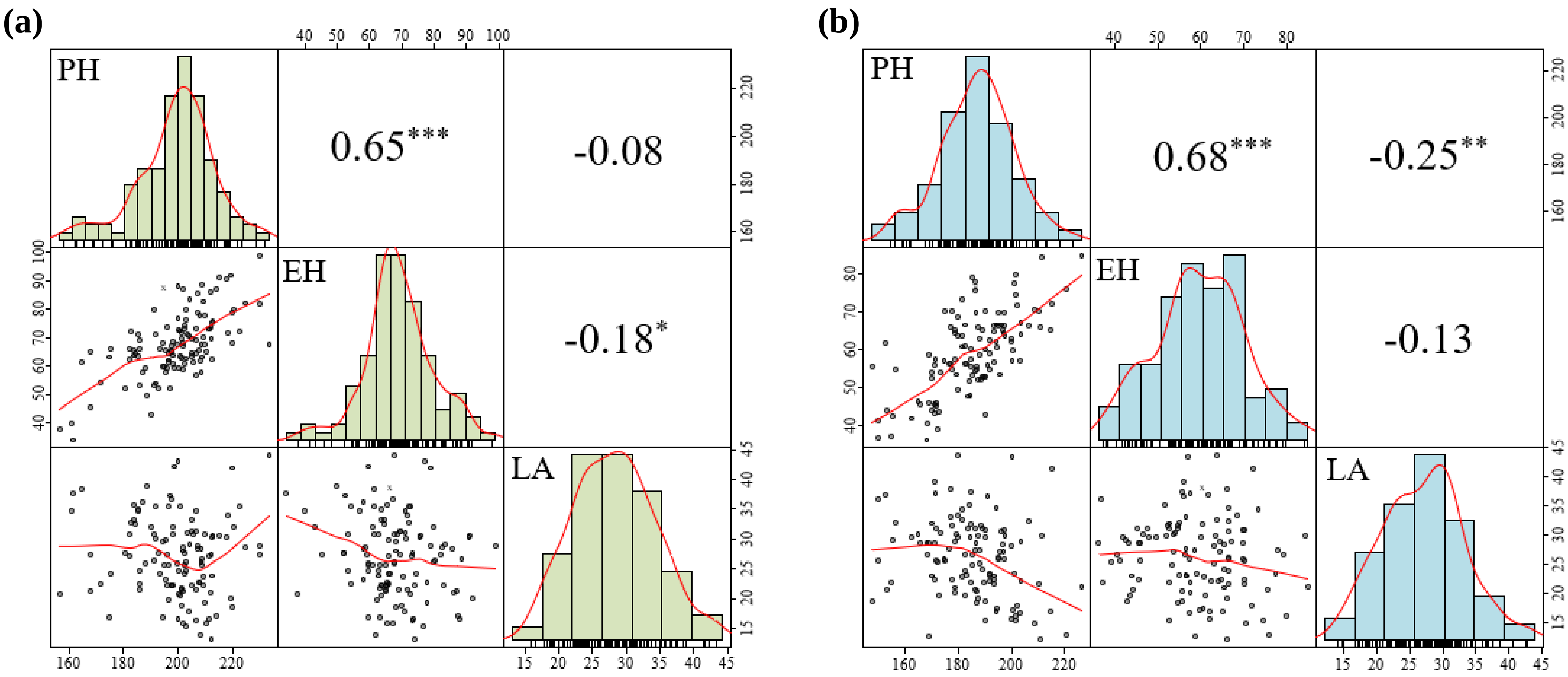

Figure S3. Correlation coefficients analysis of three traits in a maize BC_1F_4 population across two years 2016 (a) and 2017 (b). PH for plant height; EH for ear height; LA for leaf angle. The histograms on the diagonal show the phenotypic distribution of three traits. The plots below the diagonal are scatter plots of compared traits, and the values above the diagonal are the pairwise Pearson's correlation coefficients between the traits. *significant at $P < 0.05$; **significant at $P < 0.01$; ***significant at $P < 0.001$.
