# Supplementary material for: All-in-one sequencing: an improved library preparation method for cost-effective and high-throughput next-generation sequencing
Source: Plant Methods. 2020 May 24;16:74. doi: 10.1186/s13007-020-00615-3 (PMC7247233; doi:10.1186/s13007-020-00615-3)
Supplement: Supplementary file 17 — Additional file 17: Method S1. Protocol for the AIO-seq method. [file 13007_2020_615_MOESM17_ESM.doc]

**[Method S1]**

**Protocol for the AIO-seq method**

1. **DNA quantification**

The ideal range of the genomic DNA (gDNA) concentration is 5 ~ 10 ng/μL. Dilute the DNA using the Qubit^TM^ dsDNA HS Assay kit (Invitrogen, NY, USA, Cat.Q33216) as per the manufacturer’s instructions, and then determine the concentration using the Qubit^TM^ 4.0 Fluorometer (Invitrogen, NY, USA) if initial gDNA concentration is too high. It is critical to assay the entire batch of DNA samples with the same Qubit^TM^ buffer mixture to avoid concentration deviations.

1. **Library preparation**

Library preparation in the preliminary and subsequent large-scale parallel experiments was achieved using the TruePrep® DNA Library Prep Kit V2 for Illumina® (Vazyme Biotech, China, Cat. TD501-02). To further decrease the costs of the library preparation for large cohorts of samples, home-made Tn5 transposase according to the expression and purification described in previous research [1,2] could be used. Since the DNA fragment distribution pattern in the final library depends on the stoichiometry of the DNA and transposase complex, and a good DNA fragment distribution pattern will allow for even sequence data distribution among the multiplexed libraries easily, it is worth doing a preliminary experiment using approximately 3 ~ 5 DNA samples to determine the best stoichiometry of the gDNA and transposase complex, before large-scale experiments.

**2.1 A preliminary experiment to explore the best** **stoichiometry of DNA and transposase complex.**

2.1.1 Prepare the tagmentation reaction in 200 μL PCR tubes, according to Table 1, and mix gently by pipetting up and down approximately 10 times.

Table 1 Tagmentation reaction system for preliminary experiments

| Reagent | Volume (μL) | Note |
| --- | --- | --- |
| gDNA | m | the input gDNA of m μL should be 20 ~ 50 ng |
| ddH_2_O | 40-m-n |  |
| 5 × TTBL | 10 |  |
| TTE Mix V50 | n | n = 2.0, 3.0, 4.0, 5.5, 6.0 μL transposase complex for each sample |
| Total | 50 |  |

2.1.2 Cover the PCR tube, and pulse-spin to get all the solution to the bottom of the tube, and incubate the tube in a T100TM Thermal Cycler (BioRad, CA, USA) for 5 minutes at 55 ℃, then hold at 10 ℃.

2.1.3 Clean the tagmented DNA by column-based Cycle-Pure Kit (Omega Bio-Tek, GA, USA Cat.D6492-02*) according to the manufacturer’s protocol and elute the DNA twice with 20 μL of elution buffer to maximize the DNA recovery.

2.1.4 Assay the purified DNA concentration by Qubit^TM^ dsDNA HS Assay kit, usually, about 70 ~ 80 % of the input gDNA will be recovered.

2.1.5 Assay the tagmented DNA size distribution pattern with an Agilent 2100 Bioanalyzer (Agilent Technologies, CA, USA) with a High Sensitivity Kit (Agilent Technologies, CA, USA, Cat.5067-4626) or Fragment Analyzer ^TM^ (Agilent Technologies, CA, USA) with High Sensitivity NGS Fragment Analysis Kit (Agilent Technologies, CA, USA, Cat.DNF-474) according to the manufacturer’s protocol. To get the best resolution of the fragment distribution, load 2 ~ 3 ng of DNA into the Agilent 2100 Bioanalyzer or Fragment Analyzer.

2.1.6 The majority of the DNA fragments are in the range of 200 ~ 1000 bp and show an approximate trapezoidal distribution pattern. Usually, in comparison to a bell-shaped curve, the better a trapezoidal distribution pattern in tagmented DNA fragments, the better the stoichiometry of the gDNA and transposase complex.

**2.2 Large-scale parallel experiments for library preparation**

2.2.1 Prepare the tagmentation reaction in a 96-well PCR plate according to Table 2, and mix gently up and down with a pipette approximately 10 times.

Table 2 Tagmentation reaction system for large-scale experiments

| Reagent | Volume (μL) | | | | | Note |
| --- | --- | --- | --- | --- | --- | --- |
|  | Sample 1 | Sample 2 | Sample 3 | … | Sample n |  |
| gDNA | y_1_ | y_2_ | y_3_ | ... | y_n_ | The input gDNA of y μL should be 20 ~ 50 ng |
| ddH_2_O | 20-y_1_-x | 20-y_2_-x | 20-y_3_-x | ... | 20-y_n_-x |  |
| 5 × TTBL | 5 | 5 | 5 | ... | 5 |  |
| TTE Mix V50 | X | x | x | ... | x | Volume needed here is calculated according to the optimal stoichiometry of DNA and transposase complex |
| Total | 25 | 25 | 25 | … | 25 |  |

2.2.2 Cover the PCR plate, centrifuge for 1min at room temperature and then incubate in a T100^TM^ Thermal Cycler for 5 minutes at 55℃, then hold at 10℃.

2.2.3 After the tagmentation reaction, 1 μL of 2.6% SDS is added into each well to strip the Tn5 transposase within the TTE Mix V50 and it is then mixed gently with a pipette up and down, approximately 10 times, followed by 5 min of rest.

2.2.4 Prepare the PCR reaction according to Table 3, and mix gently by pipetting up and down approximately 10 times, followed by centrifuging for 1 min to get all of the solution to the bottom of the well.

Table 3 PCR reaction components

| Reagent | Volume (μL) |
| --- | --- |
| Tagmentation products with SDS | 26 |
| 5 × TAB | 10 |
| PPM | 5 |
| N5×× | 4 |
| N7×× | 4 |
| TAE | 1 |
| Total | 50 |

2.2.5 PCR enrichment was performed on T100TM Thermal Cycler with a 105℃-heat lid, according to the procedure described in Table 4.

Table 4 PCR enrichment procedure

| Temperature (℃) | Time | Cycle Number |
| --- | --- | --- |
| 72 | 3 min |  |
| 98 | 30 sec |  |
| 98 | 15 sec | 5 ~ 6 |
| 60 | 30 sec |
| 72 | 1 min |
| 72 | 5 min |  |
| 4 | Hold |  |

2.2.6 After amplification, the PCR products of each sample were purified with 1.8 × VAHTS^TM^ DNA Clean Beads (Vazyme Biotech, Nanjing, China, Cat. N411-02) and eluted in 20 μL (or less) of sterile ddH_2_O, to get the final library.

**3. Quality control of libraries**

3.1 The initial concentration of each library was read using the Qubit^TM^ 4.0 Fluorometer.

3.2 Assay the size distribution and the proportion of target regions (420 ~ 520 bp for pair-end 150 bp sequencing) for the whole library with the Agilent 2100 Bioanalyzer, Qsep100^TM^ (BiOptic, Taiwan, China) or Fragment Analyzer ^TM^ (Agilent Technologies, CA, USA).

**4. Library pooling**

Table 5 provides an example of six samples that are pooled together in a group. Briefly, samples 2 ~ 6 are expected to get even data yields, and the data yield of sample 1 is expected to be twice that of samples 2 ~ 6. So, the proportion of expected data yield in this group is 2/7 for sample 1 and 1/7 for each of the remaining 6 samples. Accordingly, the “Mass of mixed target region” is 40.0 ng for sample 1 and 20.0 ng for each of the remaining 6 samples, and the subsequent “Vol. for mixing” for each sample could be calculated with the “Mass of mixed target region” divided by TRC, where TRC could be calculated by initial “Con. of lib” multiplied by the “Ratio of 420 ~ 520 bp”. They should be mixed in a tube to get 52.29 μL of mixture. Both “Mass of mixed target region” and “Vol. for mixing” could be proportionately scaled up or down.

Table 5 Library pooling of six samples using AIO-seq

| Sample ID | Con. of lib (ng/μL) | Ratio of 420 ~ 520 bp (%) | Target region concentration (TRC, ng/μL) | Proportion of expected data yield in group | Mass of mixed target region (ng) | Vol. for mixing (μL) | Expected data yield (Gb) |
| --- | --- | --- | --- | --- | --- | --- | --- |
| 1 | 21.0 | 11 | 2.31 | 2/7 | 40.0 | 17.32 | 20.0 |
| 2 | 25.6 | 13 | 3.33 | 1/7 | 20.0 | 6.01 | 10.0 |
| 3 | 19.6 | 13 | 2.55 | 1/7 | 20.0 | 7.85 | 10.0 |
| 4 | 20.4 | 12 | 2.45 | 1/7 | 20.0 | 8.17 | 10.0 |
| 5 | 24.8 | 13 | 3.22 | 1/7 | 20.0 | 6.20 | 10.0 |
| 6 | 24.2 | 12 | 2.90 | 1/7 | 20.0 | 6.74 | 10.0 |
| Total | – | – | – | 1 | 140.0 | 52.29 | 70.0 |

**5. Size selection**

5.1 Add 5 μL of 6 × TriTrack DNA Loading Dye（Thermo Fisher Scientific, MA, USA, Cat. R1161）into the above 52.29 μL of mixture and then mix gently by pipetting up and down approximately 10 times, followed by pulse-spinning to get all the solution to the bottom of the tube.

5.2 Use the SageELF electrophoresis system (Sage Science, MA, USA) and a 2% pre-cast agarose gel cassette (Sage Science, MA, USA, Cat. ELD2010) to fractionate the 57.29 μL mixture into 12 fractions, and then run with the time-based mode following the manufacturer’s procedures. Usually, the total volume for loading into the sample well of the cassette is supposed to be less than 65 μL and the total mass should higher than 500 ng.

5.3 Absorb 12 fractionated fragments from each recovered well to 1.5 ml tubes, separately.

**6. Quality control of fractionated fragment**

6.1 Read the concentration of each fractionated fragment using the Qubit^TM^ 4.0 Fluorometer.

6.2 Use the Agilent 2100 Bioanalyzer to analyze the quality of the fractionated fragments.

**7. Quantify the selected fragment and sequencing**

Choose one or two fragment(s) with well-constrained size ranges that are in the target region (420 ~ 520 bp) for further quantitative PCR (qPCR), and then perform paired-end 150 bp high throughput sequencing on the Illumina® HiSeq X Ten system.

**References**

1. Hennig BP, Velten L, Racke I, et al. Large-scale low-cost NGS library preparation using a robust Tn5 purification and tagmentation protocol. G3-Genes Genom Genet. 2018;8:79-89.
2. Picelli S, Bjorklund AK, Reinius B, et al. Tn5 transposase and tagmentation procedures for massively scaled sequencing projects. Genome Res. 2014;24:2033-2040.
